# Observations on the Case of Mr. Windham

**Published:** 1810-08

**Authors:** George Nesse Hill

**Affiliations:** Chester


### *To the Editors of the Medical and Physical Journal.*

Gentlemen,

MEDICAL teachers very properly urge upon their audiences, the utility of reviewing every case of importance which has been committed to their care, after it has arrived at its termination; which scrutiny, if fairly conducted, must always

 be be beneficial to the individual who makes it, and ultimately to the public. Such a member of society as the late Mr. Windham cannot be removed to the "house appointed for all living," without exciting attention to the circumstances which led to the solemn change particularly when those circumstances do not rank with those of every day's occurrence.

Newspaper details cannot from their very nature be upon all occasions correct, but I take it for granted that when a number of them concur in certain leading points, it will be allowed that the truth is promulgated. What they have given the world respecting the last illness and death of the great politician we have just lost, appears to me of much importance, especially to that part of the rising generation who are qualifying for medical practitioners; if you, Gentlemen, view the subject in the same light, you will oblige me by inserting these remarks in your next Journal I feel the less reluctance in obtruding myself upon the medical public on this occasion, from the conviction that the faculty generally acknowledge the imperious necessity of medical reform; whenever this great work is *properly* attempted, it will be found indispensibly necessary to direct a portion of attention "to the artificial division of the healing art into the medical and surgical departments;" for, as it is well observed by that eminent surgeon and acute writer, Mr. Abernethy, "An evil seems to me to have arisen from this division; it has caused the attention of the physician and surgeon to be too exclusively directed to those diseases which custom has arbitrarily allotted to their care."

Mr. Windham's case (if truly given) highly illustrates the inestimable advantages of general medical knowledge when brought to the test with talents, however great, that are bounded by the too long prevailing absurd division of the healing art. We are told that this gentleman was in his 61st or 63d year; that he received a blow on the hip whilst in a state of violent bodily exertion and mental anxiety, and, as the event proved, in a state of highly unfavorable predisposition, confirmed by the observation in the report, "his powers were consumed." From all these circumstances the adoption or avoidance of the knife became a very critical point indeed for professional men to decide, who were not most intimately acquainted with the exact state of the physical condition of the subject demanding such decision. Well and truly might they say, "that the knife was at all times to be used with awe, and never but where life was absolutely at stake, or where the probable prolongation of of life was to be made endurable by relief from pain." Though separately consulted, a majority concurred "that there was no danger in avoiding the operation, but much in undergoing it," for the tumour was no serious inconvenience, and scarcely accompanied with pain." But other *surgeons* gave him an opinion that the tumour might be safely cut out, and he instantly resolved on the experiment; resolved on making his life the test of experiment: nor would he wait for any preparation of the system; a refusal he would never have made, had a degree of medical sagacity existed in the minds of some of the practitioners consulted, sufficient to point out the indispensable necessity of such a preliminary step to this and nearly every other surgical operation; but as Lord Milton observes in his eulogy, "nothing could swerve or bias him in the opinion he once had formed". How much those who esteemed him, have to lament that such firmness (as it is termed) did not receive the most rational bias at first, there is too much cause to apprehend from the whole narrative, that his active mind was not clearly directed to the primary consideration, namely, his general health, previous to and at the time of receiving the blow; also from that event to the period of consultation. Had he been sufficiently impressed with the solemn, irrefutable truth, that such a view and strict inquiry was to determine the just treatment of his case, and that the accidental tumour and its local management were mere secondary considerations, it is probable the ultimate event might have been somewhat different; for what will not an ardent mind rightly directed perform? unless it be fact, as stated, "his powers were consumed;" then indeed it is needless to add a word on the consequences of a severe manual operation, although performed with the "most perfect skill," as doubtless this was.

But it may be unadvisedly said, the tumour was the cause of all the physical disturbance, and nothing short of its immediate removal could afford the sufferer a hope of recovery; but is or can such a case be made out? Has the event, upon the enjoined review of the case now it is come to pass, justified the treatment? When are we required by imperious unavoidable necessity, to remove tumours "that give no serious inconvenience, and are scarcely accompanied with pain?" From various external causes, parts have doubtless become extraneous, and from inattention in the sufferer, or defective *medical* ability in the surgeon, they finally require removal, or nature will be as it were teased to death in her unavailing efforts to free herself from the incumbrance; incumbrance; but then the accompanying general symptoms are sufficiently unambiguous to all who devote attention to them, and by no means such as we are told (if correctly stated) existed in Mr. Windham. In his case the blow could only be the simple exciting cause, acting upon unfavourable physical condition, to produce one of those " non-descript diseases or varieties of local disease," absolutely in its origin, symptoms, progress, and termination, dependent upon the constitution and general disturbance of the health, which can produce " so many varieties, and even every variety," and upon which, medical sagacity, judiciously exercised, will on almost every occasion, be as competent as it is necessary to decide.

Now, when the result of that decision is perempt, " that there is no danger in avoiding a manual operation, but much in undergoing it," is the honour of a noble science, like that of medicine, to be committed by the obstinacy of any energetic, resolute man, be his rank what it may? There is no doubt but thousands of medical practitioners daily resist the dangerous importunities of ignorance or ill directed courage, much to the advantage of their patients and the glory of our art, never losing sight of the wholesome precept, " stand in due awe of the knife; it is not to be used but when life is absolutely at stake." *Their* language to Mr. W. would doubtless have been, the blow being received during an unfavourable state of the system, caused a greater or less degree of comminution and destruction of vessels, with a rude shock to the subjacent bone; rest, and the efforts of nature alone, in a firm healthy physical condition, have been equal to the counteraction of serious consequences from far more extensive local mischief than this; but we find such not to have happened in your case, consequently you are not in that healthy, physical condition you should be; still do not suffer a knife to approach this swelling, but attend to your general health; mark the result of the vigilant use of medicine and general means; if you do any thing externally, by way of aid to these means, apply mild soothing fomentations, absolute rest, gentle frictions promotive of absorption, leeching, &c. Should these means fail, and your " powers are consumed," they will at least be a test of the fact, and prove that the knife may hasten the termination, but cannot possibly prolong your existence; if from other advisers, aided by your own daring impatience, you are determined on an operation, and its consequences, we remonstrate, we combine against such a rash, unqualified, unjustifiable determination, determination, because you are not in a physical state to undergo it, because the mere surgical operation is the dernier, not the primary resource of our art, never to be adopted but from necessity and in doubtful or ambiguous cases with awful reluctance; but when decidedly indispensible with calm courage and well grounded hope: in your instance no such necessity exists, therefore we protest against any such a dreadful perversion of the views and resources of so incomparably valuable a branch of science as that of medicine.

I am, &c.

GEORGE NESSE HILL.

*Chester, July* 13, 1810.

